# Complete smokefree policies in mental health inpatient settings: results from a mixed-methods evaluation before and after implementing national guidance

**DOI:** 10.1186/s12913-018-3320-6

**Published:** 2018-07-11

**Authors:** Lisa Huddlestone, Harpreet Sohal, Claire Paul, Elena Ratschen

**Affiliations:** 1 Department of Epidemiology and Public Health, University of Nottingham, City Hospital, Hucknall Road, Nottingham, NG5 1PB UK; 2 Leeds and York Partnership NHS Foundation Trust, Becklin Centre, Alma Street, Leeds, LS9 7BE UK; 3 Department of Health and Social Sciences, University of York, Heslington, York, YO10 5DD UK

**Keywords:** Tobacco control, Smokefree policies, Mental health, Health inequalities

## Abstract

**Background:**

Tobacco smoking is extremely prevalent in people with severe mental illness (SMI) and has been recognised as the main contributor to widening health inequalities in this population. Historically, smoking has been deeply entrenched in the culture of mental health settings in the UK, and until recently, smokefree policies tended to be only partially implemented. However, recent national guidance and the government’s tobacco control plan now call for the implementation of complete smokefree policies. Many mental health Trusts across the UK are currently in the process of implementing the new guidance, but little is known about the impact of and experience with policy implementation.

**Methods:**

This paper reports findings from a mixed-methods evaluation of policy implementation across 12 wards in a large mental health Trust in England. Quantitative data were collected and compared before and after implementation of NICE guidance PH48 and referred to 1) identification and treatment of tobacco dependence, 2) smoking-related incident reporting, and 3) prescribing of psychotropic medication. A qualitative exploration of the experience of inpatients was also carried out. Descriptive statistical analyses were performed, and the feasibility of collecting relevant and complete data for each quantitative component was assessed. Qualitative data were analysed using thematic framework analysis.

**Results:**

Following implementation of the complete smokefree policy, increases in the numbers of patients offered smoking cessation advice (72% compared to 38%) were identified. While incident reports demonstrated a decrease in challenging behaviour during the post-PH48 period (6% compared to 23%), incidents relating to the concealment of smoking materials increased (10% compared to 2%). Patients reported encouraging changes in smoking behaviour and motivation to maintain change after discharge. However, implementation issues challenging full policy implementation, including covert facilitation of smoking by staff, were reported, and difficulties in collecting relevant and complete data for comprehensive evaluation purposes identified.

**Conclusions:**

Overall, the implementation of complete smokefree policies in mental health settings may currently be undermined by partial support. Strategies to enhance support and the establishment of suitable data collection pathways to monitor progress are required.

**Electronic supplementary material:**

The online version of this article (10.1186/s12913-018-3320-6) contains supplementary material, which is available to authorized users.

## Background

Rates of smoking among people with severe mental illness (SMI) are two to three times higher than among the general population and can reach up to 70% among hospitalised mental health patients [1]. Consequently, smokers with mental illness experience significant reductions in life expectancy due to smoking-related morbidity and mortality, with an average of 17 years of life lost to prematurely to conditions that are caused or exacerbated by smoking [2]. Due to metabolic interactions between hydrocarbon agents within tobacco smoke and the human liver enzyme CYP2A6, smokers with mental illness can require up to double the dosage of psychotropic medication to reach a therapeutic range; monitoring and adjustment of dosages are necessary for certain medications to prevent potential medication toxicity, for example in the case of the atypical antipsychotic clozapine [1]. Despite evidence that people with mental illness are similarly motivated to quit to the general population [3] and can successfully do so when provided with evidence-based support [4], tobacco use within inpatient mental health settings is historically and culturally deeply embedded [1, 5]. Addressing smoking among mental health patients has been identified as a long neglected area, and as essential in efforts to reduce health inequalities in this disadvantaged population [1]. Although mental health settings in England became ‘smokefree’ by law in July 2008, meaning that any smoking indoors was prohibited after that date, research identified the persistence of smoking as the norm in the context of blanket exemptions that were granted for patients to smoke in courtyards or other outdoor spaces on mental health Trust premises [6]. The provision of staff-supervised regular ‘smoking breaks’ for patients was estimated at an annual cost of between £18,250 and £86,870, per ward dependent upon the seniority of staff [7, 8]. Additionally, there was an indication that the facilitation of regular smoking breaks within mental health settings might increase incidents of challenging behaviours among patients and could potentially lead to increased use of prescribed pro re nata (‘as needed’) medication administration [8].

In 2013, the UK National Institute of Health and Clinical Excellence (NICE) published public health guideline 48 (PH48) for smoking cessation in secondary care, acute mental health, and maternity settings [9]. The guidance recommends the implementation of completely smoke free hospital sites without exemptions, comprehensive policies that promote and support smoking cessation and temporary abstinence, and the development of integrative treatment pathways for tobacco dependence. Implementation across mental health Trusts in England is currently underway; the government’s Tobacco Control Plan for England 2017 [10] sets the achievement of guidance implementation by the end 2018 as a target for all mental health Trusts. In view of the historic ‘smoking culture’ in mental health settings [5, 11] that involves complex psychosocial dynamics between patients, and patients and staff, and the powerful links between smoking and mental illness [1, 12], the guidance acknowledges various challenges to be likely to arise in the context of policy implementation and states the importance of monitoring outcomes in this regard. Although many mental health Trusts across England have implemented NICE guidance PH48 since its publication or are in the process of doing so, little is known so far about implementation impact and experiences in England. This paper presents the findings of a mixed-methods evaluation prior to and following the implementation of NICE PH48 in a large Northern NHS mental health Trust. Specifically, the evaluation aimed to:

1. Assess the completeness of recording of smoking-related data, including smoking status and smoking cessation/abstinence treatment offered and received;
2. Investigate potential changes in smoking-related incidents reported prior to and following smokefree policy implementation;
3. Compare average doses of psychotropic medication affected by tobacco smoking for inpatients before and after smokefree policy implementation;
4. Explore inpatients’ experience with the smokefree policy and its impact on smoking behaviour and on intentions relating to smoking after discharge following smokefree policy implementation;
5. Review the availability of relevant and complete data for the purposes of evaluation, and develop recommendations.

## Methods

### Study design

The mixed-methods evaluation consisted of two parts: 1) the collection and comparison of quantitative data, measuring patients’ smoking status and smoking-related treatment recordings, smoking-related incident reporting, and antipsychotic medication prescribing pre- and post policy implementation; and 2) a qualitative exploration of inpatient experience following smokefree policy implementation, using face-to-face interviews, after policy implementation.

### Setting and participants

The evaluation was set within a large mental health and learning disability NHS Foundation Trust in the North of England, providing acute inpatient treatment on twelve mental health inpatient wards (five acute wards, two rehabilitation wards, two dementia wards, two older adult wards, and a peri-natal ward), located across three sites and housing 156 patients in total. Prior to the implementation of NICE PH48, the Trust smoke free policy permitted regular escorted smoking breaks on-site for patients detained under the Mental Health Act, in designated outdoor smoking areas. For non-detained patients and those granted Section 17 leave under the *Mental Health Act 1983*, unescorted smoking breaks were permitted off the Trust site. Following NICE guidance PH48, the Trust revised its smokefree policy to prohibit smoking anywhere on the Trust premises, and to discontinue the escorting of detained patients to spaces where they could smoke. No exemptions to the smokefree policy were to be allowed. The policy stipulated to record all patients’ smoking status on admission, and to offer Nicotine Replacement Therapy (NRT) and behavioural support to help smokers abstain and manage their withdrawal symptoms during the hospital stay, using an in-house ‘Health and Wellbeing Service’ with trained smoking cessation advisors.

### Procedures

#### Data collection and analyses

Quantitative measures were collected over a period of two months prior to the implementation of NICE guidance PH48 (April 2016), in February and March 2016, and again over a period of two months following guidance implementation, in May and June 2016. While the pre- and post-evaluation design was chosen in line with a common methodological approach in the relevant literature [13], the data collection periods of two months before and after implementation reflected a pragmatic choice made in light of a number of constructions arising in the context of this pragmatic project. The collected data included recordings of: 1) basic demographic patient data and patient smoking status on admission; 2) delivery of brief advice and treatment (including NRT) for temporary abstinence and smoking cessation; 3) prescriptions of psychotropic medications whose metabolism is known to be significantly affected by tobacco smoking/quitting, and the prescribed regular and pro re nata (‘as needed’) dosages among patients identified as smokers; and 4) smoking-related incidents. In addition, we endeavoured to collect additional data not routinely collected on patients’ cigarette consumption on admission and at discharge (to identify changes in smoking behaviour during the admission period) on two pilot wards. The assessment of feasibility to collect relevant and complete data for each of the quantitative components took place throughout the study for each quantitative component and is reported in the relevant sections. Qualitative data relating to patients experience after smokefree policy implementation were collected in the summer of 2016. Further methodological detail relating to data collection for each study element is given below.

#### Identification and treatment of tobacco dependence

Anonymised demographic data, primary mental health diagnosis, legal status (i.e. voluntary admission or detained under the Mental Health Act), length of admission, and smoking status information were obtained from electronic patient records for all patients admitted to the 12 participating wards between 01 February and 30 March 2016 for the pre-implementation phase, and between 01 May and 30 June 2016 for the post-implementation phase. Smoking status, if recorded, was defined on the electronic Trust system as ‘current’, ‘former’ ‘never’, or ‘unknown’. Where patients indicated they had been smokers on admission, information regarding the delivery of smoking cessation advice, offers of treatment for tobacco dependence, and the type of treatment offered, were recorded. Data were coded and entered in IBM Statistical Package for Social Sciences (SPSS) and descriptively analysed for frequencies, proportions, and means including standard deviations (SD).

#### Prescribing of psychotropic medication and nicotine replacement therapies

A data set comprising prescriptions of psychotropic medications most notably influenced by smoking and smoking cessation [1] was created based on prescription transcripts for patients admitted for the pre- and post- implementation periods. Medication doses and frequency of administration were recorded in IBM SPSS version 22 for each evaluation period. Prescribed daily doses, prescribed four-weekly doses, and maximum daily pro re nata doses of medication were determined for each patient. Data were aggregated to calculate the mean dose for each main type of psychotropic medication prescribed during the pre-PH48 implementation period and the post-PH48 implementation period across all participating wards. Standard deviations were calculated where appropriate. In view of the non-normal distribution, non-parametric Mann-Whitney U tests were used to measure the direction and significance of changes in doses between the pre- and post-PH48 implementation periods. Statistical significance was defined as *p* < 0.05. In addition, data on prescribed NRT were compiled from a review of patient pharmacy records pre- and post- implementation. Frequencies, proportions, and median (including interquartile range) dosages are presented. Meaningful statistical comparison of the pre- and post-implementation data was not possible due to the scarcity of NRT prescription data.

#### Smoking-related incidents

Data relating smoking-related incidents for each ward were obtained from the Trusts electronic incident reporting system, *Datrix*, for the pre-implementation period and the post-implementation period and transferred into Microsoft Excel for data management. Searches were made using smoking and incident-related key words and their synonyms [8]. The narrative accompanying each retrieved incident report was reviewed to ensure its relevance and those reports identified as not relating to smoking were excluded from analysis. A coding framework was developed in which to record relevant manifest content. Manifest content is ‘overtly presented and quantifiable’, as opposed to latent content that requires interpretation of underlying surface level data [14]. Manifest content analysis allows for the broad observation of themes throughout a large data sample, and uses quantitative analysis to identify trends. An initial structure was established from a priori knowledge of the topic; further thematic codes were added to the coding frame as they emerged from readings of the incident reports. Relevant manifest content of each smoking related incident report were recorded using the coding frame. Data were analysed in Microsoft Excel. Descriptive statistics were used to obtain frequencies and proportions.

Levels of incident severity were defined by the Trust reporting system as 0 = no harm caused; 1 = low severity (resulting in increased patient observation or minimal changes to care plans); 2 = moderate severity (short-term harm caused); and 3 = high severity (longer-term harm caused). Data contained within each incident report were reviewed and a rating of harm applied to the incident. Data were analysed in Microsoft Excel and frequencies and proportions obtained.

#### Patients’ experience with smokefree policy implementation

An opportunistic approach to participant recruitment was adopted for the semi-structured face-to-face interview study, guided by patients’ mental health status and their interest in taking part. Patients from all participating wards, who reported to be current smokers on admission, and who were able to provide informed consent, were identified in liaison with the multi-disciplinary teams in the week prior to discharge and eligibility was confirmed with patients’ care teams. Potential participants were initially approached by a member of their care team with study information. Interviews were conducted in a private area of the ward with informed written consent. Separate consent was obtained for the audio recording of the interview and where consent was refused in-depth notes of participant responses were made by the researcher. Demographic patient data were collected and a schedule of topics guided interview discussions (Additional file 1), focussing on patients’ past and present smoking behaviours, their perceptions of a smokefree inpatient stay, and intentions relating to smoking behaviour after discharge, while allowing novel themes to emerge during the conversation.

Accounts were generated from participants using conversational prompts. Summation was employed during discussion to ensure researcher understanding and to provide the opportunity for participants to correct any accounts, thus improving accuracy and validity. Demographic data were coded and entered into Microsoft Excel and analysed descriptively for frequencies and proportions. Narrative data were transcribed verbatim and analysed using a thematic framework approach [15]. Transcripts were read repeatedly. A priori and emergent points were developed into a framework from which codes were derived and applied to the data. In order to establish coherence of the framework, interview transcripts were read and coded independently by a second researcher (LH). Where minor differences in relation to coding arose, these were settled through discussion.

## Results

### Patient characteristics

Patient characteristics for both the pre- and post-implementation cohorts are presented in Table 1.

**Table 1:** Patient characteristics, by cohort

Characteristics		Pre-implementation (*n* = 150)	Post-implementation (*n* = 165)
		*Frequency (%)*	
*Gender*	*Male*	83 (55.3)	81 (49.1)
	*Female*	67 (44.7)	84 (50.9)
*Legal status*	*Detained*	81 (54.0)	108 (65.6)
	*Voluntary*	69 (46.0)	57 (34.4)
*First admission*	*Yes*	43 (28.7)	77 (46.7)
	*No*	107 (71.3)	88 (53.3)
		*Mean (SD)*	
*Age (years)*		47.9 (20.9)	48.2 (19.8)
*Length of admission (days)*		23.2 (15.9)	37.2 (22.8)

### Identification and treatment of tobacco dependence

#### Recording of patients’ smoking status

Among the 150 patients admitted for treatment of acute mental illness during the pre-PH48 implementation period, 141 (94.0%) were asked about smoking status on admission, and from the 165 patients in the post-PH48 implementation phase, 139 (84.2%) received similar enquiries. Table 2 details the smoking status of patients for both the pre- and post-PH48 implementation periods. We were unable to collect additional information on patients’ cigarette consumption after admission and at discharge to assess changes in smoking behaviour during admission, as these non-routine data were not recorded on the wards as planned. Therefore, we were unable to to present appropriate data relating to this outcome in the context of this evaluation.

**Table 2:** Recording of smoking related information during the pre- and post PH48 implementation periods

Smoking status recorded	Frequency (%)	
	*Pre-PH48 (n = 150)*	*Post-PH48 (n = 165)*
*Current smoker*	70 (46.7)	65 (39.3)
*Former smoker*	18 (12.0)	20 (12.1)
*Never smoker*	23 (15.3)	23 (13.9)
*Unknown*	30 (20.0)	31 (18.8)
*Information refused*	1 (0.7)	3 (1.8)
*Unrecorded*	8 (5.3)	23 (13.9)

#### Characteristics of smokers identified

Among the 70 (49.6%) smokers identified during the pre-PH48 period, 44 (62.9%) were male and 26 (37.1%) were female and had a mean age of 37.4 (SD 12.84) years. Almost two-thirds of smokers (*n* = 42, 60.0%) were detained for treatment under the Mental Health Act. Fourteen (20.0%) patients had previously experienced admission for treatment of mental disorder and the mean duration of admission was 23.0 (SD 15.73) days. During the post-PH48 implementation period, among the 65 (46.7%) identified smokers, 38 (58.5%) were male and 27 (41.5%) were female with a mean age of 43.3 (SD 14.79) years. Over three-quarters (*n* = 50, 76.9%) of patients were detained under the mental health act and 24 (36.9%) had previously experienced admission for treatment of acute mental disorder. The mean duration of admission was 34.9 (SD 20.6) days.

##### Recording of brief advice and treatment for smoking cessation/temporary abstinence

During the pre-PH48 implementation period, 57 (38.0%) of the 70 current smokers received advice relating to smoking, and of these, under a third (*n* = 21, 30.0%) were offered an intervention to support cessation or temporary abstinence. Interventions offered to smokers included: prescription of NRT (*n* = 5, 23.8%) or referral to external stop smoking services (*n* = 16, 76.2%). Following implementation of PH48, 47 (72.7%) of the 65 smokers identified received advice, and of these, 59.1% (*n* = 39) were offered treatment for tobacco dependence through the in-house Health and Wellbeing service. The majority (*n* = 38, 97.4%) of patients who received an offer of treatment were recorded as being provided with NRT.

#### Prescriptions of nicotine replacement therapies

During the pre-implementation period, pharmacy records showed that five patients (7% of smokers identified) had received prescriptions of NRT. Four were prescribed nicotine patches providing a median dose of 21 mg (IQR 12.7–21.0 mg) and one patient received combination NRT comprising a 21 mg nicotine patch and a 15 mg inhalator. During the post-implementation period, 38 patients (58% of smokers identified) were recorded as having been being provided with NRT in patients’ notes, although NRT prescriptions could be identified only for 11 (28.9%) of those.. Six (54.4%) received a nicotine patch providing a median dose of 21 mg (IQR 19.5–25.0 mg). Three (27.3%) patients were prescribed a 15 mg nicotine inhalator, and two (18.2%) patients received combination NRT comprising a 21 mg patch and a 15 mg nicotine inhalator. The numbers were too small to perform meaningful statistical analysis.

#### Psychotropic medication prescribing

Substantial difficulties were encountered in collecting the relevant data for this study component. The challenges arose from the circumstance that medication data were not available electronically, and that storage locations for paper case notes and availability of authorised staff to liaise in retrieving notes varied greatly. Despite persistent attempts to retrieve the information, only partial data sets could be developed after extracting information from paper case notes: for the pre-implementation period, 141 out of 150 (94%) medication transcripts were analysed. For the post-implementation period, only 82 out of 165 (49.7%) of medication transcripts could be retrieved before the end of the project. Results of the analysis below should be considered with this limitation in mind.

Seventy-six of 141 admitted patients (53.9%), for whom medication transcripts could be retrieved, had been prescribed psychoactive drugs significantly affected by components of tobacco smoke during the pre-implementation period. The majority (*n* = 59, 77.6%) of these medications were antipsychotics; 18 (23.7%) were benzodiazepines; and 14 (18.4%) were antidepressants. Data relating to the post-implementation period identified 40 of 82 patients (48.8%), for whom records were retrieved, who were prescribed relevant psychoactive drugs. Two-thirds (*n* = 27, 67.5%) of these medications were antipsychotics; ten (25.0%) were antidepressants; and five (12.5%) were benzodiazepines. Mean daily dosages, and where appropriate in the case of longer acting drugs (indicated by an asterix), four weekly doses for each individual medication, were calculated with standard deviations (SD) (Table 3).

**Table 3:** Aggregated doses for patients’ prescribed relevant psychotropic medication by PH48 implementation period

Medication	Pre-PH48		Post-PH48	
	*n = 76*	*Mean dose (SD)*	*n = 40*	*Mean dose (SD)*
*Amitriptyline*	–	–	2	20.0 mg (0.0)
*Chlorpromazine*	–	–	2	12.0 mg (0.0)
*Clozapine*	2	325.0 mg (106.1)	4	281.2 mg (132.5)
*Diazepam*	8	21.9 mg (32.7)	4	7.6 mg (3.3)
*Duloxetine*	2	120.0 mg (0.0)	2	90.0 mg (0.0)
*Flupentixol*	2	6.0 mg (4.2)	–	–
Flupentixol Deconate*	2	260.0 mg (197.9)	–	–
*Haloperidol*	7	11.3 mg (7.2)	4	9.1 mg (5.6)
Haloperidol Depot*	3	116.6 mg (28.9)	3	133.3 mg (28.9)
*Lorazepam*	7	1.1 mg (0.47)	5	1.1 mg (0.54)
*Mirtazapine*	12	37.5 mg (10.1)	9	29.4 mg (14.5)
*Olanzapine*	37	10.2 mg (5.6)	19	9.1 mg (4.8)
*Tamazepam*	1	40.0 mg (−)	–	–
*Zuclopentixol*	5	110.0 mg (82.2)	4	54.7 mg (41.8)
Zuclopentixol Deconate*	6	1050.0 mg (234.5)	5	1600.0 mg (489.9)

* denoting longer acting medications

Non-significant decreases were identified in the dosages prescribed for the anti-depressant Mirtazapine, for the benzodiazapine diazepam, and for the antipsychotics clozapine, duloxetine, haloperidol, olanzapine, and zuclopentixol, and a non-significant increase was found in the prescribing of the longer acting injectable haloperidol depot. Mann-Whitney tests found that the aggregated dosage of the antipsychotic zuclopentixol deconate increased significantly over the post-PH48 period (U = 27.0, *p* = 0.030).

### Smoking related incidents

#### Quantitative content analysis

Smoking-related incidents were retrieved and categorised for both the pre- (*n* = 52) and post-PH48 (*n* = 394) implementation periods (Table 3). Incidents for both periods were reviewed together in order to develop categories of incident type resulting from or related to tobacco use. Four categories were identified: 1) illicit smoking; 2) concealing paraphernalia and other policy breaches, 3) wider safety concerns; and 4) challenging behaviours.

##### Illicit smoking

Twenty-eight (53.8%) incidents for the pre-PH48 period reported illicit smoking indoors. Twenty-one (75.0%) of these incidents occurred in relation to patients smoking in bedrooms; three (10.7%) were related to patients smoking in bathrooms; and four (14.3%) concerned patients smoking within shared ward areas, such as kitchen areas. The majority (*n* = 322, 81.7%) of incidents during the period following the implementation of PH48 pertained to illicit smoking, over half (*n* = 168, 52.2%) indoors: 83 (49.4%) occurred in patients’ bedrooms; 52 (31.0%) took place in patients’ bathrooms; and 33 (19.6%) were in relation to patients smoking in shared areas of the ward. One-hundred and fifty-four (47.8%) of incidents were recorded as smoking in the outside space on Trust premises. Fifty-one (33.1%) incidents of smoking by patients escorted by staff on the TTrust site were recorded and 103 (66.8%) incidents occurred when patients were unaccompanied. The majority (*n* = 92, 89.3%) of outside patient smoking was identified as taking place within the wider grounds of the TTrust site. Smoking within secure outside ward spaces (such as internal courtyards) was documented in eleven (10.7%) reports.

##### Concealing smoking paraphernalia and other policy breaches

Prior to the implementation of PH48, one (1.9%) incident was recorded describing an episode of concealment of tobacco by a patient when returning from leave. Following the implementation of PH48, almost 10% (*n* = 39) of all smoking-related incidents were concerned with concealment. Almost all (*n* = 38, 97.4%) of these related to patients concealing tobacco or smoking paraphernalia. Fifteen (39.5%) concealments were identified in patients’ bedrooms; 13 (34.2%) patients were witnessed concealing tobacco within the grounds of the unit; seven (18.4%) patients were reported as attempting to conceal tobacco and/or lighters about their person; and concealed lighters were found within the general areas of the ward on three (7.9%) occasions. The one (2.6%) remaining policy breach incident related to a visitor providing a patient with pouches of tobacco.

##### Wider safety concerns

Ten (19.2%) of the 52 recorded incidents prior to the implementation of PH48 related to ‘safety’ in the sense of having potential impact on patient and staff safety. Six (60.0%) related to patients going missing/absconding from the ward in order to smoke or to purchase tobacco without leave. Four (40.0%) further incident reports documented the use of electricity by patients as an ignition source for the lighting of cigarettes. Following the implementation of PH48, eight (2.0%) incidents relating to safety were identified. These comprised the use of electricity as a source of ignition, used to light cigarettes (*n* = 4, 50.0%) and patients absconding from the ward in order to acquire or smoke tobacco (*n* = 4, 50.0%).

##### Challenging behaviour

In the pre-implementation period, twelve (23.2%) incidents were identified as concerning challenging behaviours resulting from the use of tobacco or smoking-related arrangements within the wards. These incidents comprised reports of: physical violence by patients against staff (*n* = 7, 58.4%); verbal aggression directed towards staff (*n* = 3, 25.0%); and damage to Trust property (*n* = 1, 8.3%). One (8.3%) incidence of patient self-harm was also reported, as a result of being denied a ‘smoking break’. Following implementation of PH48, challenging patient behaviours accounted for approximately 6% (*n* = 23) of tobacco related incidents. These included: physical violence towards staff by patients (*n* = 5, 20.0%); verbal aggression directed at clinical staff by patients (n = 5, 20.0%); damage to Trust property (*n* = 7, 28.0%); harassment or bullying between patients (*n* = 4, 16.0%); patient harassment of staff (*n* = 1, 4.0%) and patient harassment of visitors (*n* = 1, 4.0%).

##### Severity of harm resulting from smoking-related incidents

Levels are harm resulting from smoking related incidents were calculated for both periods. Among the reports documenting smoking-related incidents during the pre-PH48 period, the majority (*n* = 45, 86.5%) resulted in no harm being caused; five (9.6%) reports were classed as low severity, indicating that minimal harm was caused; and one (1.9%) incident of physical violence against staff was recorded as moderate in severity, resulting in short-term harm, requiring medical treatment. The great majority (*n* = 378, 95.9%) of the incidents reported during the post-PH48 period were rated as resulting in no harm being caused to staff, patients, or the general public, while 16 (4.1%) incidence were classed a ‘low’ severity, indicating that minimal harm resulted. No moderate or severe cases of harm were identified.

### Patient experience

#### Participant characteristics

Nine patients were recruited over several months from the participating rehabilitation and acute adult mental health wards. Recruitment in this study population and setting was challenging and occurred in liaison with wards staff; a formal record to document recruitment rates was not kept. Of the nine recruited patients, eight consented to audio recording; in the remaining case, detailed notes were taken by the interviewer. Interviews lasted between 30 and 40 min. Six (66.7%) participants were male and three (33.3%) were female. The mean age was 32.6 (SD 5.81) years. Four reported a primary diagnosis of schizophrenia, one of bipolar disorder, and one of psychosis; the remaining three diagnoses were undisclosed or unknown. Two participants had quit smoking since admission to the inpatient ward, and all others, with one exception, reported to smoke significantly less than they had before admission. Those who still smoked consumed between five and 40 cigarettes daily [mean 16.0 (SD 10.50)]. Motivation to make a quit attempt among the seven smokers was reported with a mean rating of 4.3 (SD 2.82) from a maximum of ten points on a visual analogue scale. Confidence in successfully achieving cessation, if a quit attempt was made, was rated at a mean of 6.2 (SD 2.72) from a maximum of ten points.

#### Thematic framework analysis

Interview data were analysed with reference to pre-identified topics and emerging themes and grouped into four main thematic areas: 1) past and present smoking behaviours: adjusting to the smokefree policy; 2) policy realities: enforcement and support; 3) challenges related to cessation and abstinence; and 4) motivation to quit and thoughts on smoking after discharge. As anticipated in this patient population, the flow of conversations and depth of data gathered during the interviews varied, with some communication problems being apparent in some interviews. The analysis below should be viewed as indicative of relevant content and as preliminary, suitable to inform further exploration.

##### Theme 1: Past and present smoking behaviour: Adjusting to the smokefree policy

Most interview participants described taking up smoking early in life, often prompted by peer-pressure or even facilitated by their family. With one exception, all reported having attempted to quit smoking at least once in their lives previously, with most having succeeded for at least brief periods of time. Notably, seven of nine participants had either quit or reduced their smoking as a result of admission to a smokefree ward: two patients reported complete abstinence with only rare lapses at the time of interview, and six reported reduction of consumption, three of whom substantial, from over 20 per day to less than five. Two patients reported smoking more than they used to, due to the fact that during periods of leave from the wards more than one cigarette in a short period of time.

“*Because it’s illegal here, smoking, on your leave you feel like need to smoke fast, fast, fast, fast* [in case] *something happens.*” (Participant 1: Male, acute ward).

“*I only get eight half an hour a day, unescorted* [leave]. *I usually smoke two when I am out…That’s because of the leave I need to make sure I keep my nicotine levels up. Having the one cigarette now is not enough, I’ve got to have two.”* [Participant 4, Male; Rehabilitation ward].

Several participants reported the use of a range of mostly single NRT products to help manage their abstinence, while others highlighted their belief in ‘going cold turkey’ using ‘will power’, and one highlighted the use of electronic cigarettes as an aid to maintain abstinence. Despite some critical views, patients appeared overall accepting of the policy.

*‘I thought it [smokefree policy] is never going to work, but it…, I suppose it’ll have to work, there is no excuses now, is there.’* [Participant 5: Male; Acute ward].

‘*It gets easier as time goes along and that, but I’d like to quit fully and that lot, but because of the stress you go through in here, you need something.’* [Participant 6: Female; Acute ward].

One participant reported that his clozapine dose had been reduced by 50% since he quit smoking on admission: *‘If I smoke it’s about 400 ml, because I’ve stopped smoking they’ve reduced it to 200 ml, they keep count of me, is it white cells, and have a look at my white cells and my diabetes’* [Participant 2: Male; Rehabilitation ward].

##### Theme 2: Policy realities: Adherence, enforcement and support

Many participants described utilising their leave entitlement to smoke covertly within the grounds of the unit, viewing them as ‘smoking breaks’. Participants reported developing strategies to negotiate nicotine withdrawal, with the majority reporting smoking a number of cigarettes while on leave, in order to counter the periods of time that they are unable to smoke. Furthermore, the concealment of tobacco and smoking paraphernalia was reported by participants to be common practice, and one which is recognised and even supported by some staff members.

*“Within 24 hours of me being here, I was advised by the staff, by one member of staff, to ‘find a stash’ for my cigarettes outside… I appreciated him doing it.”* [Participant 4: Male, Rehabilitation ward].

*‘I stashed the lighter outside because we are not allowed lighters in the building, so I have never, ever smoked in the building, erm, every time I go on leave on me own, I nip to a spot where, which I know and that and I have a 2 cigarettes at a time, and I come back in, erm, that’s it really.’* [Participant 3: Female; Acute ward].

*“You’ve only got to walk out at any point in the day and someone is fiddling about in the trees or the bushes for the* cigarettes” [Participant 8; Male; Rehabilitation ward].

One patient however described a different experience of strong policy enforcement that was perceived as inappropriate:

“*Some people have a stick rammed right up their backside where they’re challenging patients. To me that’s not a way to be with people with mental illness…They do that in front of everybody instead of taking them to one side”* [Participant 4; Male; Acute ward].

In terms of support, there was evidence of staff offering patients NRT products on admission, but no accounts relating to the offer or uptake of behavioural support or any information relating to the links of smoking with medication were elicited. While some patients reportedly used NRT, they conveyed a sense of a lack of more comprehensive support following initial discussions with staff after admission concerning abstinence support: for example “*They just said, “We can offer you some chewing gum” and that was about it.”* [Participant 5; Male; Acute ward].

Some patients described witnessing staff smoking on Trust premises, alone or with patients:

*‘I’ve seen at least five cleaners smoking, I’ve seen three staff, one from this ward and a couple from another… I’ve even seen them smoking with patients as well, so they’ve taken somebody for a walk, and they sit and smoke together.’* [Participant 7; Female; Rehabilitation ward].

##### Theme 3: Challenges to maintaining abstinence and achieving cessation

Two participants cited boredom and stress as challenges to managing their cravings and maintaining abstinence on the wards. Furthermore, there was a notion that a lack of knowledge and information with regard to strategies to support stopping smoking, especially the use of NRT products, prevailed. Despite all patients receiving a brief smoking cessation intervention upon admission many participants displayed a number of misapprehensions regarding the use of NRT, resulting in some choosing not to use the products offered. For example, one participant claimed “*When I have a patch or nicotine, it puts nicotine in my system and it makes me want more”* [Participant 2; Male; Rehabilitation ward], and several were relying on will power to manage their cravings alone. There was a lack of understanding that electronic cigarettes are substantially less harmful than tobacco smoking, with one participant stating that *‘nobody really knows yet how harmful it is for* you’ [Participant 3; Female; Acute ward].

Clearly, there was an awareness of covert smoking taking place on Trust premises despite the policy, much by way of representing an implicitly accepted norm rather than an exception, as demonstrated in previous quotes. Essentially, it became clear during the interviews that the new policy could only be considered partial.

##### Theme 4: Motivation to quit and thoughts on smoking after discharge

Many participants stated they wanted to quit smoking entirely or maintain the reduction of consumption they had achieved during the inpatient stay after discharge. Some expressed positive notions of the idea to access stop smoking services or harm reduction measures in the community following discharge: “*I would like go to a smoke-free clinic*” [Participant 4:Male, Acute ward]. For some, electronic cigarettes were considered an acceptable cessation method with one participant stating “*An e-cig would stop me from smoking for life”* [Participant 2: Male; Rehabilitation ward]. One participant highlighted the importance of her son’s approval of her quitting smoking as a motivator to keep up a smokefree lifestyle after discharge: ‘*The fact that my son is over the moon that I’ve stopped smoking…that’s enough for me [to stay smokefree]*’ [Participant 7; Female; Rehabilitation ward].

Drinking coffee and alcohol and watching movies in which characters smoked were cited by some participants as potential trigger for relapse when returning home. Several participants mentioned that the use of cannabis presented a trigger for smoking relapse:

*‘Yeah, well now, I’ve been stopping smoking but I know when I get out I’ll probably smoke a spliff, so I might end up back on cigarettes…’* [Participant 2: Male; Rehabilitation ward].

Notably, one participant reported a lack of encouragement from community health professionals in relation to smoking cessation in the context of mental illness: “*If you go to the doctors to ask help for stop smoking while you’ve got mental health issues, they tell you to sort out your mental health issues first*” [Participant 3: Female, Acute ward]. Some participants mentioned that further information on how to stop smoking, face-to-face and group support would help them quit. One stressed that receiving a reward, for making a successful quit attempt would be an incentive for her: ‘*For me to stop smoking, there has to be a reward at the end of it, do you know what I mean? … Some kind of special award… I don’t know, like a certificate or something.*’ [Participant 3; Female; Acute ward].

## Discussion

This mixed-methods evaluation demonstrates that there are notable challenges in collecting relevant and complete data for a comprehensive evaluation of smokefree policies in mental health settings that are likely to affect mental health Trusts across the country. Confident interpretation of the quantitative evaluation results is therefore in parts limited, especially where changes in psychotropic medication prescribing are concerned. Qualitative results indicate the emergence of a number of implementation issues that have the potential to result in serious undermining of the policy, with likely adverse effects on patients and staff. Despite this, the qualitative data highlighted overall encouraging changes in smoking behaviour as well as high motivation to maintain or advance this change after discharge, in line with emerging literature [16]. The need to secure enhanced staff and patient support and to establish meaningful smoking-related data collection pathways is apparent.

### Supporting patients who smoke

While the proportion of patients who received recorded enquiries in relation to smoking status decreased slightly in the post-PH48 implementation period, the recording of the delivery of brief advice, offers, and uptake of treatment for tobacco dependence increased considerably, which should be interpreted as encouraging. However, although more patients were provided with NRT according to case note records, observed rates of NRT prescriptions within pharmacy records appeared to be low, although this could at least be partly explained by the incompleteness of the pharmacy data set we were able to obtain. Still, both quantitative and qualitative findings indicate that the prescribing of combination NRT, as recommended for the support of heavily dependent smokers [1], is uncommon.

In the absence of information relating to changes of patients’ smoking status and/or smoking behaviour following admission, it appears challenging to analyse and interpret the impact of smokefree policy implementation in a meaningful and comprehensive way. Furthermore, failing to review and discuss patients’ smoking at discharge may mean that opportunities for supporting patients after discharge to reduce their tobacco consumption or encourage quit attempts may be missed [17]. Recent research has shown that post-discharge cessation support is effective in motivating quit attempts and reductions in cigarette consumption for up to six months post-discharge [18]. In line with NICE [9] and other [2] recommendations, future research should consider how best to integrate post discharge support for tobacco dependence into community mental health services.

### Policy adherence and enforcement

One of the most unexpected and salient insights from this project pertained to the apparent emergence of practices that resulted in smoking being yet again established as a ‘norm’ – despite the implementation of a complete smokefree policy. Patients in our study reported that the grounds of the site provided numerous opportunities to hide tobacco and smoke covertly. Accounts of stashed cigarettes and paraphernalia, of regular smoking on Trust premises during leave from the wards, and of staff facilitating and sometimes participating in smoking bear close resemblance to findings from research conducted after the first implementation of smokefree policies in mental health settings in 2008 [6, 19], and before NICE guidance PH48 implementation [8]. This research concluded that the establishment of implicit pathways that facilitate smoking results in a number of adverse effects. In line with findings from the international literature [5, 20–22], the partial rather than comprehensive smoke free policy appeared to limit effectiveness in promoting and supporting cessation or temporary abstinence among patients and the culture of tobacco use. It was also clear that misconceptions related to the use of NRT and electronic cigarettes prevailed, and that the recollection and uptake of offers of behavioural support was limited.

### Smoking-related incidents

Our evaluation contained an analysis of all events classed as ‘incidents’ according to Trust policies and thus went further than previous studies, which focused on incidents involving violence and aggression only [23]. Unsurprisingly, our analysis identified an increase in reported illicit smoking following smokefree policy implementation compared to pre-implementation. However, in common with international studies investigating the establishment of smokefree mental health environments, it showed that challenging patient behaviours, in particular violent incidents involving staff, decreased after the implementation of PH48 [23–27]. As discussed by Robson et al. in their recent study in a UK mental health Trust [23], this is an important finding, seeing as concerns over potential increases in violence are cited as one of the main reasons for mental health Trust’s reluctance to implement complete smoking bans rigorously. It is also in line with conclusions from international reviews [20, 22], showing that the commonly anticipated surge in violent incidents did not transpire. It is worth noting that in our study, recorded incidents of damage to property increased from 8 to 28% during the study period. The reasons for this are unclear and could be related to a purely descriptive analysis, which did not account for potential confounders as other studies with more complex analysis techniques [23], or to enhanced recording practice following implementation. Continuous monitoring of smoking-related incidents should be established as an indicator of smokefree policy implementation progress.

### Patient experience and needs

Although some criticism of the policy was expressed by participants in our study, patients reported they were coping with it well – though in many cases admittedly making use of opportunities to smoke covertly on outdoor Trust premises. Nevertheless, in line with other emerging evidence [16], a number of participants in our study reported that admission to a smokefree hospital hadencouraged contemplation of quitting, and that two of nine previously smoking participants were abstinent from tobacco at the time of interview. In line with the evidence [3, 4], this challenges the widely held tacit assumption that patients with mental health problems are less interested in or less able to quit smoking and highlights the great potential of a successfully implemented smokefree policy and the importance of pursing this goal. Based on our quantitative findings, anecdotal concerns in relation to patients seeking early discharge, at times against clinical advice, appear unfounded.

A number of factors considered likely to trigger relapse to smoking after discharge, including the use of cannabis, were mentioned by participants, and overall acceptance expressed towards the notion of accessing smoking cessation support, with preferences of types of support varying. The need therefore to develop and test support pathways for patients who smoke following a smokefree inpatient stay is evident. This is in line with studies from the US and Australia that highlight the potential of post-discharge smoking cessation interventions in the community [18, 28].

Participants reported misapprehensions and uncertainty of the efficacy of the support available. This highlights the need to further understand patients’ preferences for preparing for admission to a smokefree setting, and the provision of NRT to support temporary abstinence. Furthermore, the need to increase the capacity of staff to effectively identify and address gaps in patient knowledge is highlighted. The management of nicotine withdrawal should be proactive, with an understanding of evidence-based strategies (including use of of combination therapies and supporting temporary abstinence), knowledge of the potential of electronic cigarettes as harm reduction aids in this population [29]. The delivery of smoking cessation interventions requires staff to acquire skills and knowledge to both understand the issues and apply a managed intervention to address both withdrawal and dependence. Furthermore, reference to boredom and stress *(‘you need something’*) emphasizes the importance of offering therapeutic activities appropriate to support smoking abstinence, in addition to comprehensive evidence-based cessation and abstinence support.

### Challenges, strengths and limitations

The findings of this study should be interpreted in light of several limitations. As indicated previously, we encountered substantial difficulties in collecting adequate data for some of the study components, especially the component related to potential changes in antipsychotic medication. We interpret these difficulties as indicative of challenges likely to arise in the context of future evaluations and research studies in this area and feel strongly that the development and maintenance of data collection pathways suitable to review progress in this area should be highlighted. This is also true for the availability of smoking-related data that would enable interpretation of policy effects in a more comprehensive way. For example, we were unable to collect any structured information on changes in patients’ smoking behaviour post discharge: smoking status information was only collected on admission, and no further information, e.g. usefully on cigarette consumption by the time of discharge, was recorded. In the absence of such data, interpretation relating to the effects of smokefree policy implementation is difficult. During the project, we endeavoured to initiate recording of such data on two pilot wards. This failed due to non-compliance of staff, resulting in a pilot data yield that was substantially too small to be considered for analysis.

Recruitment of participants for the qualitative interviews proved challenging in this population and took place opportunistically (in line with qualitative methodology) over several months. It is possible that the experiences of those patients who were willing to talk to us are not generalizable to all smokers in the study setting. However, despite potential concerns of internal and external validity, important unexpected themes emerged in unison across accounts, enabling a deeper understanding of processes and underlying issues that quantitative data alone would not have provided.

Another limitation arises from a relatively small sample of wards from one single Trust, and also from the relatively short periods of time data were collected pre- and post- implementation (2 months, respectively) that were chosen pragmatically – in close proximity to implementation itself. Long-term assessments of policy impact, supported by appropriate data collection pathways, are required to complement short-term findings.

Nonetheless, to our knowledge, this is the first evaluation of its kind in England, and despite the limitations constitutes a valuable addition to the scarce evidence in this area, highlighting some encouraging results as well as emerging implementation issues. We are confident that many of the issues raised here will be relevant for other mental health Trusts in the country, and hope that our findings will provide a starting point for the development of further data collection and open discourse in this area.

## Conclusion

Suitable data collection pathways for the meaningful evaluation and interpretation of impacts related to smokefree policy implementation in mental health settings are crucial and currently not in place. There is an indication and a concern that efforts to implement comprehensive policies could be significantly undermined by the establishment of tacitly accepted covert smoking rules. The importance of continuous monitoring of processes and outcomes related to the policy, of enabling staff to support patients and the policy comprehensively, and of creating inpatient environments supportive of managing tobacco abstinence, is highlighted.

## Additional file

Additional file 1: Interview schedule. (DOCX 68 kb)

## Abbreviations

NICE: National Institute for Health and Care Excellence
NRT: Nicotine Replacement Therapy
SMI: Severe mental illness
