# Supplementary material for: Complete smokefree policies in mental health inpatient settings: results from a mixed-methods evaluation before and after implementing national guidance
Source: BMC Health Serv Res. 2018 Jul 11;18:542. doi: 10.1186/s12913-018-3320-6 (PMC6042321; doi:10.1186/s12913-018-3320-6)
Supplement: Supplementary file 1 — Interview schedule. (DOCX 68 kb) [file 12913_2018_3320_MOESM1_ESM.docx]

**
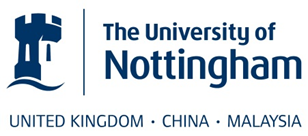
**

**Interview schedule**

**Smoke-free mental health settings: exploring service users’ experiences**

**Introduction:**

- Introduce interviewer
- Explain the aims and purpose of the study and give a brief description of the interview structure.
- Ensure Participants have read the information sheet
- Discuss digital recording of the interview and confidentiality
- Opportunity for participant to ask any question
- Complete the consent form and give a copy to participant

**Topic 1: Smoking and quitting histories**

Can you tell me more about your smoking (when started; how much; ever quit)?

**Topic 2: Smoking and addressing smoking while an in-patient**

Can you tell me about your experience of smoking and quitting since you have been admitted?

- - (Let themes emerge…)
  - Positives and negatives
  - Support offered and received (pharmacological and behavioural; how quickly?)

**Topic 3: Smoking cessation/reduction while an in-patient**

1. Has your smoking behaviour changed since your admission?
   - How?
   - Feelings about this
   - Is it something you would like to maintain?
2. Could you tell me about any experiences of using smoking cessation as an in-patient?
   - What products
   - Single or multiple types?
   - Likes or dislikes
   - Would you consider using these products after discharge?

**Topic 4: Harm reduction**

1. Have you ever tried e-cigs?
   - If so, what type do you use?
   - Why do you use them?
   - What are the positives of e-cigs?
   - What are any negatives about e-cigs?
   - If no, why have you chosen not to use them?

**Topic 5: Support needs**

1. If you were to quit or reduce your smoking, what do you believe would help you to maintain your attempt?
   - (Text messages, talking to your relatives about your smoking Phone call from advisor?)
2. If you were to stop smoking while an in-patient, what do you feel would support you to stay smoke free once you were discharged from hospital?
   - Any particular strategies
   - Any products, support materials, or persons
3. Who do you feel would be a useful source of support in keeping you smoke-free after discharge?

- Why do you believe that?

1. Are there any things which might make you want to smoke when you return home?
   - What are they
   - Why do you believe they may trigger you to smoke?

*In the extremely unlikely event of a participant becoming distressed, disruptive or aggressive, the researcher will pause the interview and ask the participant if they feel that they would like to continue with the interview. Should they agree sufficient time will be allowed for the participant to regain their composure. If the participant declines continuation of the interview, they will be offered the opportunity to withdraw temporarily (with a new date/time arranged) or completely (in which case data will be deleted).*

*In the case of disclosure which presents risk to the participant or anyone else, the participant will be made aware of the researcher/interviewers’ duty of care and a discussion will take place as to the way to proceed. This may mean direct reporting to the PI or safeguarding officer or in cases with less risk advice of places to seek support may suffice.*

**Short Debrief:**

The interviewer will now explain the interview is now officially over and there are no more questions. They will state when the project will be ending and that if after this date, it gets published that we will let them know. The volunteers will be thanked for their participation, and asked if they would like to have a more in depth debrief, for example if what has been discussed has made them feel particularly emotional. Even if they decline the debrief at the time, it will be reinforced that we can arrange for one if on reflection they feel they would like to talk to someone. The interviewer will ensure that participants are not left distressed, and we can signpost them to individuals with expertise in this topic area if they require extra support. Participants will be reminded that while they are a patient on the ward, they have access to smoking cessation support and treatment from a member of their care team, if they choose to. Participants will also be given a separate information sheet with contact details of their local stop smoking support service in the community.
